# Quality of initial anticoagulant treatment and risk of CTEPH after acute pulmonary embolism

**DOI:** 10.1371/journal.pone.0232354

**Published:** 2020-04-28

**Authors:** Gudula J. A. M. Boon, Nienke van Rein, Harm Jan Bogaard, Yvonne M. Ende-Verhaar, Menno V. Huisman, Lucia J. M. Kroft, Felix J. M. van der Meer, Lilian J. Meijboom, Petr Symersky, Anton Vonk Noordegraaf, Frederikus A. Klok

**Affiliations:** 1 Department of Thrombosis and Hemostasis, Leiden University Medical Center, Leiden, The Netherlands; 2 Department of Clinical Pharmacy and Toxicology, Leiden University Medical Center, Leiden, The Netherlands; 3 Department of Pulmonary Medicine, Amsterdam University Medical Centers, Amsterdam, The Netherlands; 4 Department of Radiology, Leiden University Medical Center, Leiden, The Netherlands; 5 Department of Radiology and Nuclear medicine, Amsterdam University Medical Centers, Amsterdam, The Netherlands; 6 Department of Cardiac Surgery, VU University Medical Center, Amsterdam, The Netherlands; Medizinische Universitat Graz, AUSTRIA

## Abstract

**Background:**

The pathophysiology of chronic thromboembolic pulmonary hypertension (CTEPH) is not fully understood. Poor-quality anticoagulation may contribute to a higher risk of CTEPH after acute pulmonary embolism (PE), partly explaining the transition from acute PE to CTEPH. We assessed the association between the time in therapeutic range (TTR) of vitamin-K antagonist (VKA) treatment and incidence of CTEPH after a PE diagnosis.

**Methods:**

Case-control study in which the time spent in, under and above therapeutic range was calculated in 44 PE patients who were subsequently diagnosed with CTEPH (cases). Controls comprised 150 consecutive PE patients in whom echocardiograms two years later did not show pulmonary hypertension. All patients were treated with VKA for at least 6 months after the PE diagnosis. Time in (TTR), under and above range were calculated. Mean differences between cases and controls were estimated by linear regression.

**Results:**

Mean TTR during the initial 6-month treatment period was 72% in cases versus 78% in controls (mean difference -6%, 95%CI -12 to -0.1), mainly explained by more time above the therapeutic range in the cases. Mean difference of time under range was 0% (95%CI -6 to 7) and 2% (95CI% -3 to 7) during the first 3 and 6 months, respectively. In a multivariable model, adjusted odds ratios (ORs) for CTEPH were around unity considering different thresholds for ‘poor anticoagulation’, i.e. TTR <50%, <60% and <70%.

**Conclusion:**

Subtherapeutic initial anticoagulation was not more prevalent among PE patients diagnosed with CTEPH than in those who did not develop CTEPH.

## Introduction

Chronic thromboembolic pulmonary hypertension (CTEPH) is traditionally referred to as a rare, long-term complication of acute pulmonary embolism (PE). [1] Although the exact pathophysiology is not yet completely understood, a well-accepted theory is the combination of incomplete thrombus resolution after PE and vascular remodeling in previously unaffected vessels secondary to high shear stress. [2] However and remarkably, at least 25% of CTEPH patients do not have a history of confirmed PE [3], the risk profiles for PE and CTEPH differ considerably [4], and in a majority of CTEPH patients with prior PE, signs of CTEPH were already evident on echocardiography and computed tomography pulmonary angiography (CTPA) at the time of the index PE. [5–8] This latter is suggestive of diagnostic misclassification rather than CTEPH being the consequence of poorly resolved acute symptomatic PE.

Even in this setting of potential diagnostic misclassification, according to current guidelines, a CTEPH diagnosis can only be confirmed after at least three months of effective anticoagulation, as to prevent performing pulmonary artery endarterectomy of fresh blood clots. [9] In this three-month period, high-quality initial anticoagulation should also prevent recurrent venous thromboembolism (VTE), a notable additional risk factor for CTEPH. [10, 11] In DVT, subtherapeutic initial anticoagulant treatment indeed results in poorer long-term vessel patency and is a well-known risk factor for post-thrombotic syndrome. [12] In parallel, poor-quality anticoagulation may contribute to a higher risk of CTEPH after acute PE as well, although studies focusing on this issue are currently unavailable.

We aimed to evaluate whether poor-quality anticoagulation in the first 3–6 months following a diagnosis of PE would be more prevalent in patients with confirmed CTEPH than in patients who were not diagnosed with CTEPH in the clinical course of acute PE. Such knowledge could not only help identifying a potential risk factor for CTEPH, but would also shed light on the pathophysiologic mechanism of the transition from acute PE to CTEPH.

## Material and methods

### Study population

In this case-control study, we focused on 50 consecutive patients diagnosed with and treated for CTEPH in the Amsterdam University Medical Centers (Amsterdam UMC) between 2014 and 2016. These patients (cases) had previously been diagnosed with acute PE and were included in the InShape III study, details of which have been described previously. [5] Of the 50 patients, 44 provided written informed consent and could be evaluated in the current analysis. Their CTEPH diagnosis was confirmed by right heart catheterization (RHC) and pulmonary angiography in accordance with current guideline recommendations. [9] The control group was a convenience cohort from two previous prospective studies from the Leiden University Medical Center (LUMC): 150 consecutive patients with acute PE who had an echocardiography without signs of pulmonary hypertension after a follow-up period of at least 2 years. [13–15] All study participants had been treated with vitamin K antagonists (VKA) for the index PE, preceded by unfractionated and/or low molecular weight heparin for at least five days.

Ethics approval for this analysis was obtained from the local Medical Ethics Committees from both the Amsterdam UMC and the LUMC. All CTEPH patients provided written informed consent and all control patients had provided informed consent for collection of relevant data upon inclusion of the previous LUMC studies. [13–15]

### Data collection

International Normalized Ratio (INR) measurements of the first 6 months of treatment after the index PE, or if relevant, of the treatment period following a recurrent VTE diagnosis were collected. These values were retrospectively requested from local Thrombosis Services in the Netherlands, where the study patients had been monitored regularly.

### Exposure

The exposure was the time in therapeutic range (TTR) in cases versus controls as well as the time under and above this range. We also studied whether poor anticoagulation was associated with CTEPH incidence. Patients were considered to have poor anticoagulation control in case of a TTR <60%, sensitivity analyses were performed for a TTR cutoff of 50% and 70%. [16, 17]

### Determination of time in therapeutic range

An INR value per day was assigned between two consecutive INR measurements, assuming a linear relationship according to the validated Rosendaal method. [18] The maximum time allowed between two consecutive INR measurements was set at 49 days, comprising the maximum measurement interval of 42 days according to international guidelines, with an additional margin of 7 days. [19, 20] In wider intervals, a linear increase or decrease between the two values may not be plausible, which can result in biased INR estimates. [21]

Time in, under and above therapeutic range per patient was calculated for each separate treatment period. Therapeutic range was defined as an INR ≥2.0 and ≤3.5 for the entire study population. This comprises both the current VKA intensity target according international guidelines (INR 2.0 to 3.0) [19] as well as the previous Dutch national standard (INR 2.5 to 3.5), which was held until April 2016 as decided by the Federation of Dutch Anticoagulation Clinics. [22]

### Statistical analyses

Continuous variables were reported as mean with standard deviation (SD), and categorical variables as numbers with percentages. Mean TTR values were compared across both groups for the first 3 and 6 months after treatment initiation using the Independent samples T-test. To measure the association between presence of CTEPH in patients with good versus poor anticoagulation control, logistic regression was used to estimated odds ratios (ORs) with corresponding 95% confidence intervals (95% CI). The ORs were adjusted for age, sex and type of VKA used. All statistical tests were performed using SPSS Statistics software (version 23.0, IBM) and R version 3.5.1.

## Results

### Patients

Patient characteristics at the time of first PE diagnosis (index event) are provided in Table 1. The male-female ratio was roughly 1:1 in both groups. Mean age at baseline was 60 (±15) years in cases and 48 (±15) years in controls. Index PEs were diagnosed between 1985 and 2017; 35 patients (18%) had been diagnosed with at least one VTE recurrence after the index PE diagnosis. VKA treatment consisted of phenprocoumon in 41% of cases and in 89% of controls, and acenocoumarol in 59% and 10%, respectively. Only one control patient (0.67%) was treated with warfarin. The INR target range had been 2.0 to 3.0 in 73% of cases and 94% of controls. The remaining patients had an INR target range of 2.5 to 3.5 (27% and 5.3%, respectively).

**Table 1:** Baseline characteristics.

	PE patients with confirmed CTEPH later on *(n = 44)*	PE patients (in whom CTEPH is ruled out) *(n = 150)*
*General characteristics*		
Male	22 (51)	74 (49)
Age at baseline	60 (±15)	48 (±15)
*Recurrent VTE*	21 (48)	14 (9.3)
*Vitamin K antagonist*		
Phenprocoumon	18 (41)	134 (89)
Acenocoumarol	26 (59)	15 (10)
Warfarin	0	1 (0.67)
*INR target range at baseline*		
2.0–3.0	32 (73)	142 (95)
2.5–3.5	12 (27)	8 (5.3)

Continuous variables denoted as mean (± standard deviation), categorical variables as number (percentage). Baseline is defined as the moment of first VTE diagnosis.

Abbreviations: PE, pulmonary embolism; VTE, venous thromboembolism; INR, International Normalized Ratio; CTEPH, chronic thromboembolic pulmonary hypertension.

#### Availability of INR data

INR data were available in 185 patients (95%). In the remaining patients, INR data was retrieved for the treatment period after a diagnosis of recurrent VTE that occurred between the index PE diagnosis and CTEPH diagnosis (n = 9) (Table 2). In 3 control patients, data was only available beyond the first 3 months due to long-term hospitalization during which they received either heparin intravenously and/or LMWH treatment.

**Table 2:** Mean percentages of time spent in, under and above therapeutic range and corresponding mean differences.

	Patients with CTEPH (%)	Patients without CTEPH (%)	Mean difference (95%CI) ^#^
**First available VTE: 3-month analysis** *	*n = 44*	*n = 147*	
Time under therapeutic range	13	13	0 (-6 to 7)
Time in therapeutic range	69	73	-4 (-12 to 4)
Time above therapeutic range	18	14	4 (-3 to 11)
**First available VTE: 6-month analysis** *	*n = 44*	*n = 150*	
Time under therapeutic range	12	10	2 (-3 to 7)
Time in therapeutic range	72	78	-6 (-12 to -0.1)
Time above therapeutic range	16	12	4 (-1 to 9)
**First PE only: 3-month analysis** **	*n = 35*	*n = 147*	
Time under therapeutic range	11	13	-2 (-9 to 5)
Time in therapeutic range	71	73	-2 (-10 to 7)
Time above therapeutic range	17	14	3 (-4 to 11)
**First PE only: 6-month analysis** **	*n = 35*	*n = 150*	
Time under therapeutic range	11	10	1 (-4 to 6)
Time in therapeutic range	74	78	-4 (-10 to 3)
Time above therapeutic range	15	12	3 (-3 to 8)

* Including all patients with INR data from the first available VTE.

** Including all patients with INR data available of the first episode of PE only.

^#^ Independent samples T-test.

Abbreviations: 95%CI, 95% confidence interval; VTE, venous thromboembolism; PE, pulmonary embolism; INR, International Normalized Ratio.

### Proportion of time in, under and above therapeutic range

**Fig 1:**
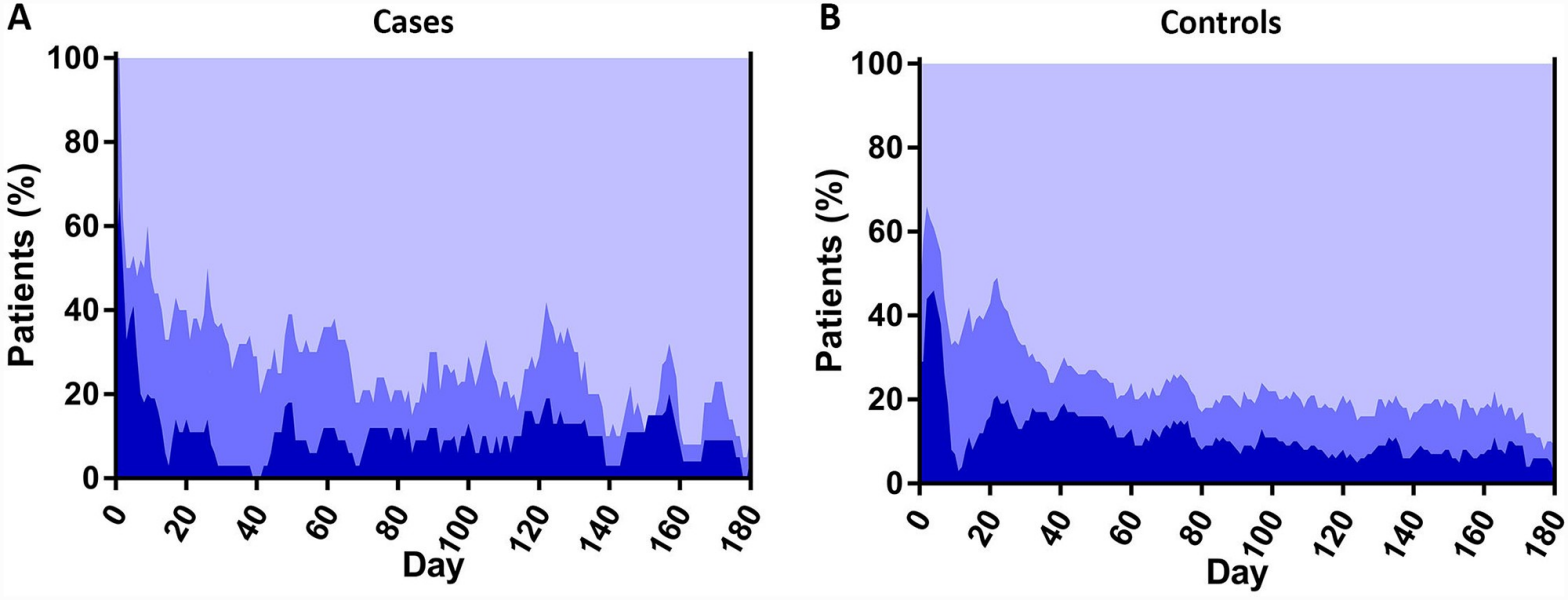
Proportion of cases (1A) and controls (1B) in, above and under INR range per day from the start of VKA therapy.

The calculated overall proportion of cases and controls in and out of the therapeutic range per day from the start of initial VKA treatment up to the 6-month of follow-up is displayed in Fig 1, demonstrating a trend towards better treatment control in controls versus cases. Mean TTR was 69% in cases versus 73% in controls, for a mean difference of -4% (95%CI -12 to 4) during the first 3 months, and 72% versus 78%, respectively (mean difference -6%; 95%CI -12 to -0.1) during the first 6 months of treatment (Table 2). This difference in TTR was mostly explained by a longer time above the therapeutic target in the CTEPH patients: after 3 months, time under therapeutic range was 13% in cases versus 13% in controls (mean difference 0%; 95%CI -6 to 7). This was 12% versus 10%, respectively, after 6 months (mean difference 2%; 95%CI -3 to 7). Time above therapeutic range was 18% in cases versus 14% in controls at 3 months (mean difference 4%; 95%CI -3 to 11), and 16% versus 12% at 6 months (mean difference 4%; 95%CI -1 to 9). Focussing on patients with INR data available after the index PE diagnosis, we found similar results: the mean TTR was 71% for cases and 73% for controls at the 3-month follow-up (mean difference -2%; 95%CI -10 to 7). This was 74% and 78% after 6 months of follow-up, respectively (mean difference -4%; 95%CI -10 to 3).

### Poor anticoagulation control and CTEPH development

A mean TTR ≤60% did not predict CTEPH, with an adjusted OR of 1.4 (95%CI 0.6 to 3.2) and 1.4 (95%CI 0.6 to 3.3) at 3 and 6 months, respectively (Table 3), nor did a mean TTR ≤50% or ≤70%.

**Table 3:** Proportion of patients under and above different thresholds of TTR and its association with CTEPH after the first available VTE.

	Patients with CTEPH (%)	Patients without CTEPH (%)	Odds ratio (95% CI)	Odds ratio (95%CI)*	Odds ratio (95%CI)**
***3-month analysis***	*n = 44*	*n = 147*			
≥ 50%	35 (80)	119 (81)	1.0 (ref)	1.0 (ref)	1.0 (ref)
< 50%	9 (20)	28 (19)	1.1 (0.5 to 2.5)	1.1 (0.4 to 2.6)	1.1 (0.4 to 3.0)
**6-month analysis**	*n = 44*	*n = 150*			
≥ 50%	39 (89)	137 (91)	1.0 (ref)	1.0 (ref)	1.0 (ref)
< 50%	5 (11)	13 (9)	1.4 (0.5 to 4.0)	1.3 (0.4 to 4.2)	0.6 (0.1 to 2.3)
**3-month analysis**	*n = 44*	*n = 147*			
≥ 60%	29 (66)	108 (73)	1.0 (ref)	1.0 (ref)	1.0 (ref)
< 60%	15 (34)	39 (27)	1.4 (0.7 to 3.0)	1.5 (0.7 to 3.3)	1.4 (0.6 to 3.2)
**6-month analysis**	*n = 44*	*n = 150*			
≥ 60%	31 (70)	126 (84)	1.0 (ref)	1.0 (ref)	1.0 (ref)
< 60%	13 (30)	24 (16)	2.2 (1.01 to 4.8)	2.1 (0.9 to 5.0)	1.4 (0.6 to 3.3)
**3-month analysis**	*n = 44*	*n = 147*			
≥ 70%	27 (61)	93 (63)	1.0 (ref)	1.0 (ref)	1.0 (ref)
< 70%	17 (39)	54 (37)	1.1 (0.5 to 2.2)	1.2 (0.5 to 2.4)	1.3 (0.5 to 2.9)
**6-month analysis**	*n = 44*	*n = 150*			
≥ 70%	25 (57)	103 (69)	1.0 (ref)	1.0 (ref)	1.0 (ref)
< 70%	19 (43)	47 (31)	1.7 (0.8 to 3.3)	1.8 (0.9 to 3.8)	1.8 (0.8 to 4.1)

^^^ Showing data of patients with an initial acute PE, of which in 9 patients INR data was only available from recurrent VTE, i.e. 6 recurrent PE and 3 DVT episodes.

*Adjusted for age and sex.

**Adjusted for age, sex and type of VKA.

Abbreviations: CTEPH, chronic thromboembolic pulmonary hypertension; VTE, venous thromboembolism; TTR, time in therapeutic range; PE, pulmonary embolism; DVT, deep vein thrombosis; VKA; vitamin K antagonist.

## Discussion

For the first time, our results demonstrate that poor initial anticoagulation control with VKA for acute PE was not more prevalent among PE patients diagnosed with CTEPH than in those who did not develop CTEPH. Notably, if anything, cases spent more time above than under the therapeutic range than the controls. Also, we did not find evidence for an association between the anticoagulation control using a fixed threshold for achieved TTR and case or control status.

We have two explanations for our findings. First, the quality of anticoagulation was high. In our study, the mean TTR was 72–78% in the 6-month analysis, whereas previous studies in VTE as well as atrial fibrillation patients, including multiple randomized controlled trials, have showed a mean TTR of 60% or lower. [12, 23] Our chosen wide therapeutic range with a higher upper limit of INR 3.5 (rather than 3.0), retrospectively applied to all individuals, has probably largely contributed to this. Consequently, because none of our study patients fulfilled the criteria of ‘poor anticoagulation’, we cannot rule out a causal association between poor anticoagulation control with VKA and CTEPH after acute PE. The other way around, although the TTR found in our study was not fully representative for daily practice, we observed that even high-quality anticoagulation did not prevent the cases from being diagnosed with CTEPH.

A second explanation could be that CTEPH might not necessarily develop after an episode of acute symptomatic PE. In fact, the International Prospective CTEPH Registry showed that only three quarters of 679 CTEPH patients had a history of confirmed acute PE. [3] Moreover, it has been demonstrated that the index CTPA used to diagnose acute PE in patients with an ultimate diagnosis of CTEPH demonstrated many radiological signs of CTEPH, i.e. intravascular webs; pulmonary artery retraction or dilatation; bronchial artery dilatation; right ventricular (RV) hypertrophy; and interventricular septum flattening. [5, 6] Notably, the presence of 3 or more of these criteria was almost diagnostic for CTEPH. [5] The diagnostic misclassification implied by this observation makes it unlikely that even high-quality anticoagulation could have prevented CTEPH since it was already present before the start of anticoagulant treatment.

Even though indefinite anticoagulant therapy in CTEPH is undisputed in current guidelines, the efficacy and safety of anticoagulant treatment in CTEPH have been poorly studied. [9] Notably, anticoagulation therapy in this setting is mostly aimed at preventing recurrent VTE and/or in situ pulmonary artery thrombosis, even after successful pulmonary endarterectomy, rather than treating CTEPH itself. [24] A potential role of poor-quality anticoagulation in the development of CTEPH after PE has been briefly suggested, for instance, in a small observational study, in which 92 PE patients were followed for 18 months through echocardiography and CTPA. All patients appeared to have had suboptimal anticoagulation therapy according to patient compliance to both therapy and monitoring regimens. Notably, TTR values were not reported. [25] CTEPH was reported in 20% of patients though they were not diagnosed according to the current standard [9], and likely represented an overestimation of the CTEPH incidence.

Importantly, VKAs are no longer the first-line treatment in VTE since direct oral anticoagulants (DOACs) feature a more favorable safety profile than VKA. [26] Our study therefore shows unique and accurate data on the quality of initial anticoagulant treatment with VKA in relation to CTEPH after PE, data that are unlikely available in recent PE or CTEPH registries. Notably, in this new anticoagulation era, TTR is no longer an issue but adherence to therapy has emerged as important new challenge for achieving high-quality anticoagulation treatment. [27, 28] It would therefore be interesting to study the prevalence of CTEPH in the years before and after introduction of the DOACs, in relation to medication adherence.

A limitation of our work is the relatively small study population, which is due to the difficulty of achieving a high number of cases on VKA treatment in the current DOAC era, as well as the low incidence of CTEPH in PE patients. As a consequence, the possible insufficient statistical power to make a definite conclusion on the association between anticoagulation quality after PE and the risk of CTEPH. Moreover, the retrospective nature of our study caused unavailability of INR data of the first VTE episode in 9 patients, which was replaced by INR data after a subsequent recurrent VTE diagnosis. Also, the target INR was not the same for all patients and controls due to local protocols and a change in national guidelines. Lastly, phenprocoumon was the choice of treatment for the vast majority in the control group, whereas acenocoumarol was more often prescribed in the cases. The choice of VKA was different between the groups due to local preferences, which could have resulted in relevant confounding with regard to the achieved quality of anticoagulation control. Of note, since the control patients were mostly treated with the more ‘stable’ phenprocoumon, this study limitation actually supports our main conclusion.

In conclusion, PE patients diagnosed with CTEPH were not found to have a higher prevalence of subtherapeutic initial anticoagulation than PE patients who did not develop CTEPH over the course of 2 years. The quality of anticoagulation was even comparable between cases and controls. An important observation is that high-quality anticoagulation did not prevent CTEPH in the patients in our study, possibly due to diagnostic misclassification of CTEPH and PE.

## Supporting information

S1 Dataset (XLSX)

## Abbreviations

95% CI: 95% confidence interval
CTEPH: chronic thromboembolic pulmonary hypertension
CTPA: computed tomography pulmonary angiography
DOAC: direct oral anticoagulant
DVT: deep vein thrombosis
INR: international normalized ratio
LUMC: Leiden University Medical Center
OR: odds ratio
PE: pulmonary embolism
RHC: right heart catheterization
RV hypertrophy: right ventricular hypertrophy
TTR: time in therapeutic range
VKA: vitamin-K antagonist
VTE: venous thromboembolism

## References

[1] Huisman MV, Barco S, Cannegieter SC, Le Gal G, Konstantinides SV, Reitsma PH, et al Pulmonary embolism. Nature reviews Disease primers. 2018;4:18028 10.1038/nrdp.2018.28 29770793

[2] Simonneau G, Torbicki A, Dorfmuller P, Kim N. The pathophysiology of chronic thromboembolic pulmonary hypertension. European respiratory review: an official journal of the European Respiratory Society. 2017;26(143). 10.1183/16000617.0112-2016 PMC9488693 28356405

[3] Pepke-Zaba J, Delcroix M, Lang I, Mayer E, Jansa P, Ambroz D, et al Chronic thromboembolic pulmonary hypertension (CTEPH): results from an international prospective registry. Circulation. 2011;124(18):1973–81. 10.1161/CIRCULATIONAHA.110.015008 21969018

[4] Egermayer P, Peacock AJ. Is pulmonary embolism a common cause of chronic pulmonary hypertension? Limitations of the embolic hypothesis. The European respiratory journal. 2000;15(3):440–8. 10.1034/j.1399-3003.2000.15.03.x 10759434

[5] Ende-Verhaar YM, Meijboom LJ, Kroft LJM, Beenen LFM, Boon G, Middeldorp S, et al Usefulness of standard computed tomography pulmonary angiography performed for acute pulmonary embolism for identification of chronic thromboembolic pulmonary hypertension: results of the InShape III study. The Journal of heart and lung transplantation: the official publication of the International Society for Heart Transplantation. 2019. 10.1016/j.healun.2019.03.003 30962147

[6] Guerin L, Couturaud F, Parent F, Revel MP, Gillaizeau F, Planquette B, et al Prevalence of chronic thromboembolic pulmonary hypertension after acute pulmonary embolism. Prevalence of CTEPH after pulmonary embolism. Thrombosis and haemostasis. 2014;112(3):598–605. 10.1160/TH13-07-0538 24898545

[7] Ende-Verhaar YM, Huisman MV, Klok FA. To screen or not to screen for chronic thromboembolic pulmonary hypertension after acute pulmonary embolism. Thromb Res. 2017;151:1–7. 10.1016/j.thromres.2016.12.026 28073030

[8] Ende-Verhaar YM, van den Hout WB, Bogaard HJ, Meijboom LJ, Huisman MV, Symersky P, et al Healthcare utilization in chronic thromboembolic pulmonary hypertension after acute pulmonary embolism. Journal of thrombosis and haemostasis: JTH. 2018;16(11):2168–74. 10.1111/jth.14266 30099844

[9] Galie N, Humbert M, Vachiery JL, Gibbs S, Lang I, Torbicki A, et al 2015 ESC/ERS Guidelines for the Diagnosis and Treatment of Pulmonary Hypertension. Revista espanola de cardiologia (English ed). 2016;69(2):177. 26837729 10.1016/j.rec.2016.01.002

[10] Ende-Verhaar YM, Cannegieter SC, Vonk Noordegraaf A, Delcroix M, Pruszczyk P, Mairuhu AT, et al Incidence of chronic thromboembolic pulmonary hypertension after acute pulmonary embolism: a contemporary view of the published literature. The European respiratory journal. 2017;49(2). 10.1183/13993003.01792-2016 28232411

[11] Palareti G, Legnani C, Cosmi B, Guazzaloca G, Cini M, Mattarozzi S. Poor anticoagulation quality in the first 3 months after unprovoked venous thromboembolism is a risk factor for long-term recurrence. Journal of thrombosis and haemostasis: JTH. 2005;3(5):955–61. 10.1111/j.1538-7836.2005.01330.x 15869591

[12] van Dongen CJ, Prandoni P, Frulla M, Marchiori A, Prins MH, Hutten BA. Relation between quality of anticoagulant treatment and the development of the postthrombotic syndrome. Journal of thrombosis and haemostasis: JTH. 2005;3(5):939–42. 10.1111/j.1538-7836.2005.01333.x 15869588

[13] Klok FA, van Kralingen KW, van Dijk AP, Heyning FH, Vliegen HW, Huisman MV. Prospective cardiopulmonary screening program to detect chronic thromboembolic pulmonary hypertension in patients after acute pulmonary embolism. Haematologica. 2010;95(6):970–5. 10.3324/haematol.2009.018960 20053871 PMC2878796

[14] van der Bijl N, Klok FA, Huisman MV, van Rooden JK, Mertens BJA, de Roos A, et al Measurement of right and left ventricular function by ECG-synchronized CT scanning in patients with acute pulmonary embolism: usefulness for predicting short-term outcome. Chest. 2011;140(4):1008–15. 10.1378/chest.10-3174 21474573

[15] Klok FA, Van Der Bijl N, Eikenboom HC, Van Rooden CJ, De Roos A, Kroft LJ, et al Comparison of CT assessed right ventricular size and cardiac biomarkers for predicting short-term clinical outcome in normotensive patients suspected of having acute pulmonary embolism. Journal of thrombosis and haemostasis: JTH. 2010;8(4):853–6. 10.1111/j.1538-7836.2010.03780.x 20096002

[16] White HD, Gruber M, Feyzi J, Kaatz S, Tse HF, Husted S, et al Comparison of outcomes among patients randomized to warfarin therapy according to anticoagulant control: results from SPORTIF III and V. Archives of internal medicine. 2007;167(3):239–45. 10.1001/archinte.167.3.239 17296878

[17] Caldeira D, Cruz I, Morgado G, Stuart B, Gomes AC, Martins C, et al Is the time in therapeutic range using the ratio of tests equivalent to the Rosendaal method? Blood coagulation & fibrinolysis: an international journal in haemostasis and thrombosis. 2015;26(8):972–6. 26083988 10.1097/MBC.0000000000000312

[18] Rosendaal FR, Cannegieter SC, van der Meer FJ, Briet E. A method to determine the optimal intensity of oral anticoagulant therapy. Thrombosis and haemostasis. 1993;69(3):236–9. 8470047

[19] Kearon C, Akl EA, Ornelas J, Blaivas A, Jimenez D, Bounameaux H, et al Antithrombotic Therapy for VTE Disease: CHEST Guideline and Expert Panel Report. Chest. 2016;149(2):315–52. 10.1016/j.chest.2015.11.026 26867832

[20] van Rein N, Lijfering WM, Bos MH, Herruer MH, Vermaas HW, van der Meer FJ, et al Objectives and Design of BLEEDS: A Cohort Study to Identify New Risk Factors and Predictors for Major Bleeding during Treatment with Vitamin K Antagonists. PloS one. 2016;11(12):e0164485 10.1371/journal.pone.0164485 27935941 PMC5147785

[21] Cannegieter SC, Rosendaal FR, Wintzen AR, van der Meer FJ, Vandenbroucke JP, Briet E. Optimal oral anticoagulant therapy in patients with mechanical heart valves. The New England journal of medicine. 1995;333(1):11–7. 10.1056/NEJM199507063330103 7776988

[22] NIV. Richtlijn Antitrombotisch beleid: Nederlandse Internisten Vereniging; [updated December 2015. https://internisten.nl/files/Richtlijn%20Antitrombotisch%20beleid_def.pdf.

[23] Baker WL, Cios DA, Sander SD, Coleman CI. Meta-analysis to assess the quality of warfarin control in atrial fibrillation patients in the United States. Journal of managed care pharmacy: JMCP. 2009;15(3):244–52. 10.18553/jmcp.2009.15.3.244 19326955 PMC10437339

[24] Pepke-Zaba J, Jansa P, Kim NH, Naeije R, Simonneau G. Chronic thromboembolic pulmonary hypertension: role of medical therapy. The European respiratory journal. 2013;41(4):985–90. 10.1183/09031936.00201612 23397304

[25] Dutt TS, Mohan BV, Tousheed SZ, Ramanjenaya R, Shetty DP. Incidence of chronic thrombo-embolic pulmonary hypertension following acute pulmonary thrombo-embolism: an Indian perspective. The Indian journal of chest diseases & allied sciences. 2013;55(4):205–7. 24660562

[26] van der Hulle T, Kooiman J, den Exter PL, Dekkers OM, Klok FA, Huisman MV. Effectiveness and safety of novel oral anticoagulants as compared with vitamin K antagonists in the treatment of acute symptomatic venous thromboembolism: a systematic review and meta-analysis. Journal of thrombosis and haemostasis: JTH. 2014;12(3):320–8. 10.1111/jth.12485 24330006

[27] Abdou JK, Auyeung V, Patel JP, Arya R. Adherence to long-term anticoagulation treatment, what is known and what the future might hold. British journal of haematology. 2016;174(1):30–42. 10.1111/bjh.14134 27173746

[28] Dronkers CEA, Lijfering WM, Teichert M, van der Meer FJM, Klok FA, Cannegieter SC, et al Persistence to direct oral anticoagulants for acute venous thromboembolism. Thrombosis research. 2018;167:135–41. 10.1016/j.thromres.2018.05.013 29843087

