# Correction: Quality of initial anticoagulant treatment and risk of CTEPH after acute pulmonary embolism

**DOI:** 10.1371/journal.pone.0234298

**Published:** 2020-06-02

**Authors:** Gudula J. A. M. Boon, Nienke van Rein, Harm Jan Bogaard, Yvonne M. Ende-Verhaar, Menno V. Huisman, Lucia J. M. Kroft, Felix J. M. van der Meer, Lilian J. Meijboom, Petr Symersky, Anton Vonk Noordegraaf, Frederikus A. Klok

The second author's initials and the seventh author’s initials are indexed incorrectly in PubMed. The correct initials for the second author are: van Rein N. The correct initials for the seventh author are: van der Meer FJM.

The second author's initials also appear incorrectly in the citation. The correct citation is: Boon GJAM, van Rein N, Bogaard HJ, Ende-Verhaar YM, Huisman MV, Kroft LJM, et al. (2020) Quality of initial anticoagulant treatment and risk of CTEPH after acute pulmonary embolism. PLoS ONE 15(4): e0232354. https://doi.org/10.1371/journal.pone.0232354.
